# Comparative Evaluation of Serum Urea, Uric Acid, and Creatine Kinase Levels in Oral Cancer and Potentially Malignant Disorders of the Oral Cavity: A Clinico-Biochemical Study

**DOI:** 10.7759/cureus.39123

**Published:** 2023-05-17

**Authors:** Lavanya Dharmana, Anupama Pottam, Sandya Rani Kollabathula, P. Suresh Kumar, Vinod Birra, Rupa Chandini Dabbiru

**Affiliations:** 1 Department of Dentistry, Andhra Medical College, Visakhapatnam, IND; 2 Department of Oral Medicine and Radiology, Anil Neerukonda Institute of Dental Sciences, Visakhapatnam, IND; 3 Department of Oral Medicine and Radiology, Gandhi Institute of Technology and Management Dental College and Hospital, Visakhapatnam, IND

**Keywords:** oral cancer, opmd, creatine kinase, uric acid, urea

## Abstract

Introduction

Evidence suggests that oral potentially malignant disorders (OPMDs) are linked to increased possibilities of malignant transformation, creating a challenging situation. If oral cancer is detected early, the prognosis is better. The purpose of this study was to compare the serum levels of urea, uric acid (UA), and creatine kinase in patients with provisionally diagnosed and histopathologically confirmed cases of potentially malignant disorders and oral cancer to those of age- and sex-matched healthy controls.

Materials and methods

Eighty patients over the age of 18 with a clinical diagnosis of oral potentially malignant disorder (OPMD) or oral cancer and verified histopathology were included in the research. Using the kinetic methodology, the enzymatic colorimetric method, and the UV-kinetic approach, respectively, the serum concentrations of urea, uric acid, and creatine kinase were quantified in vitro following the venipuncture of 2 mL of venous blood. The Statistical Package for the Social Sciences (SPSS) version 20 (IBM SPSS Statistics, Armonk, NY, USA) was used for the statistical analysis.

Results

Comparing OPMD and oral cancer patients to healthy controls, serum urea levels were found to be greater, uric acid levels to be lower, and creatine kinase levels to be higher.

Conclusion

Prognostic markers for OPMDs and oral cancer may include urea, uric acid, and creatine kinase. However, this may be accomplished by large-scale prospective research.

## Introduction

The World Health Organization (WHO) defined potentially malignant disorders (PMDs) in 2005 as conditions or lesions that may progress to cancer in the future [1]. Multiple factors may contribute to the development of cancer. By avoiding risk factors such as tobacco use (of any kind) and chewing tobacco (pan masala, khaini, and gutka) and areca nut products, oral cancers may be avoided. It is well known that alcohol and nicotine interact synergistically [2].

Oral potentially malignant disorder (OPMD) incidence rates have ranged in the Indian subcontinent from 0.6 per 1,000 people to 30.2 per 1,000 people. OPMD occurrence has been estimated to range from 0.2% to 11.3% [3]. Leukoplakia, erythroplakia, oral lichen planus (OLP), oral submucous fibrosis (OSMF), and palatal changes are all OPMDs that may occur in ex-smokers who have quit smoking [4]. People in South and Southeast Asia, especially the Indian subcontinent, are disproportionately affected by oral submucous fibrosis, a chronic, insidious disease. Over 2.5 million people in India are thought to be ill as a result of this [5]. OSMF is on the rise in India, and many believe that the country’s love of areca nuts is to blame. OSMF occurs in around 0.5% of the population [6] and has a progression to malignancy rate of 7.6% [2].

A chronic autoimmune disease of the skin and mucocutaneous system known as lichen planus may manifest in a variety of ways, including on the skin, scalp, nails, oral mucosa, and vaginal mucosa. Between 0.5% and 2% of people worldwide have lichen planus [7]. It affects 2.6% of the population in India. A 2% chance exists that lichen planus could transform into cancer [7]. The practice of switching roles while smoking is widespread along the coast of Andhra Pradesh. When compared to non-smokers, oral mucosal lesions in ex-smokers had a 19-fold higher risk of developing into malignancy, with 83.3% risk of malignancy for epithelial dysplasia and 12.5% for oral squamous cell carcinoma (OSCC) [8].

Squamous cell carcinoma (SCC) of the oral cavity is the most common kind of cancer in the head and neck region. Of all oral malignancies, oral squamous cell carcinoma (OSCC) accounts for around 92%-95% [1]. Males are affected at a rate two to three times greater than females because of their higher rates of cigarette and alcohol usage. Most oral squamous cell carcinomas are thought to originate in premalignant states [8]. Significant biochemical changes in OSMF include elevated serum copper and reduced iron. Oral cancer and leukoplakia patients often have higher serum copper levels than the general population. Patients with oral cancer, OSMF, and OLP had significantly reduced serum zinc levels. Patients with oral cancer have lower levels of iron and selenium in their bodies than healthy individuals do. Individuals with OSCC, OLP, and OSMF have been shown to have significantly higher blood lactate dehydrogenase activity. Alkaline phosphatase levels in OSCC are rising [9]. A link between OPMDs and urea, uric acid (UA), creatine kinase, as well as some cancers, has recently emerged in promising research. The estimated levels of urea, uric acid, and creatine kinase in people with OPMDs and oral cancer were compared to those in a control group by researchers. Creatine kinase, urea, and uric acid measurements can be used to diagnose OPMDs and oral cancer.

## Materials and methods

In a case-control study involving individuals with oral cancer, PMDs, and healthy controls, the levels of serum urea, uric acid, and creatine kinase were assessed. The study was conducted over the course of six months, from January to June 2018. The institution’s ethical review board approved the study and assigned it with IRB number IEC/GDCH/12/111.

Procedure

A complete medical history was taken. The type of tobacco used, amount used, length of use, and frequency of use were all entered on an authorized proforma sheet for the harmful habit history. Patients were asked questions and given a thorough physical examination in their native language. Oral and perioral regions were the focus of a thorough clinical evaluation. Two senior faculty members from the Gandhian Institute of Technology and Management Dental College and Hospital’s Department of Oral Medicine and Radiology also validated the mucosal alterations in the mouth. Study participants included patients of both sexes who had been diagnosed with oral cancer, leukoplakia, OSMF, lichen planus, or palatal changes in reverse smokers, individuals older than 18 years old, and age- and sex-matched controls. People who regularly partake in alcoholic beverages or sugary foods high in fructose, patients with weakened immune systems and those with systemic disorders, patients undergoing treatment for oral cancer and patients who have undergone radiotherapy, chemotherapy, or surgical interventions, and people who have taken medications such as diuretics, steroids, and nonsteroidal anti-inflammatory drugs (NSAIDs) in the past four weeks that could affect their serum urea, uric acid, and creatine kinase levels were excluded from the study.

Study population

A total of 120 people met the inclusion criteria. The people involved in the research were split into three categories. In group 1, 40 individuals were diagnosed clinically and tentatively as having OPMDs. There are four distinct groups within group 1. Group A consisted of 10 people who had a clinical suspicion of having leukoplakia. Ten individuals with an OSMF clinical and preliminary diagnosis made up group B. Group C consisted of 10 people who had a tentative clinical diagnosis of oral lichen planus. Ten individuals with palatal alterations after quitting smoking were included in group D. In group 2, 40 individuals with oral cancer were confirmed by clinical and histological examinations. In group 3, 40 individuals served as healthy controls with no pathological behaviors to report and no oral lesions or systemic disorders.

Two experienced professors in the fields of oral medicine and radiology took photographs of all visible mouth lesions and double-checked their accuracy. Following an examination, patients with oral potentially malignant diseases (OPMDs) underwent punch biopsies with their informed consent to confirm their diagnoses. Following up on every case that was thought to be malignant, a tissue sample was sent to the Department of Oral and Maxillofacial Pathology for a conclusive histological diagnosis. After a 12-hour fast during the night, blood was drawn from the antecubital vein using a 22-gauge needle, sterile collection containers, and a sterile environment without the use of the anticoagulant ethylenediamine tetraacetic acid (EDTA). Both the study and control groups were included in this procedure. It took the sample 30 minutes to clot. The serum was divided by centrifugation at 3,000 rpm for five minutes, aspirated into tubes, and then tested for urea, UA, and creatine kinase in a fully automated analyzer (Roche of CobasC311, Ascent Healthcare, Pune, India). The concept is based on the kinetic approach and the enzymatic end-point method.

Serum urea, uric acid, and creatine kinase levels were measured, and the results were then entered into an Excel spreadsheet (Microsoft Corp., Redmond, WA, USA) and examined using the Statistical Package for the Social Sciences (SPSS) version 20 (IBM SPSS Statistics, Armonk, NY, USA). The P-value was considered significant when it was less than 0.05. To compare continuous variables, an analysis of variance (ANOVA) was employed. Categorical variables were compared using the chi-square test. Necessary statistics such as mean, mode, standard deviation (SD), and frequency were used.

## Results

Gender-based distribution of the study population shows that in potentially malignant disorders, 20 (50%) were male patients and 20 (50%) were female patients. The oral cancer group involved 28 (70%) male patients and 12 (30%) female patients, and the control group involved 16 (40%) male patients and 24 (60%) female patients. A total of 64 (57.78%) patients were male and 56 (42.22%) were female. There were 40 patients with oral cancer, 40 patients with oral potentially malignant disorders, and 40 healthy controls (Table 1).

**Table 1:** Distribution of male and female subjects in three groups (control, malignant, and potentially malignant) Chi-square = 4.7372, P = 0.0936

Gender	Control	%	Malignant	%	Potentially malignant	%	Total	%
Male	16	40.00	28	70.00	20	50.00	64	57.78
Female	24	60.00	12	30.00	20	50.00	56	42.22
Total	40	100.00	40	100.00	40	100.00	120	100.00

The mean (±SD) age was 49.15 (±11.339) years for the 40 patients with oral cancer, 44.93 (±10.97) years for the 40 individuals with OPMDs, and 36.30 (±15.58) years for the 40 age- and sex-matched healthy controls. Subjects had a mean (±SD) age of 45.84 (±12.24). Participants in the research ranged in age from 22 to 69 years.

When intergroup and intragroup comparison was done between 40 cases of oral cancer, 40 cases of potentially malignant disorders, and 40 age- and sex-matched healthy controls between the three parameters urea, uric acid, and creatine kinase, there was a statistically significant difference in mean urea level between groups (P = 0.0013) when comparing both sets of data (Table 2).

**Table 2:** Comparison of all six groups with urea, uric acid, and creatine kinase scores by one-way ANOVA ANOVA: analysis of variance, SD: standard deviation, OSMF: oral submucous fibrosis

Groups	Urea		Uric acid		Creatine kinase	
	Mean	SD	Mean	SD	Mean	SD
Lichen planus	21.00	4.50	4.57	0.91	126.56	91.32
Leukoplakia	21.64	6.93	4.24	0.61	98.29	63.01
OSMF	20.50	5.18	4.03	0.65	95.25	63.40
Palatal changes in reverse smokers	21.00	8.91	4.40	0.56	100.25	18.87
Malignant	22.51	3.86	4.20	0.85	113.76	106.38
Control	13.90	3.18	4.71	0.69	75.70	13.07
Total	20.87	5.67	4.30	0.77	104.73	81.53
P-value	0.0013		0.2865		0.7652	

In the lichen planus group, the mean (±SD) urea score was 21 (±4.50) mg/dL, the mean (±SD) uric acid score was 4.57 (±0.91) mg/dL, and the mean (±SD) creatine kinase score was 126.56 (±91.32) U/L. In the leukoplakia group, the mean (±SD) urea score was 21.64 (±6.93) mg/dL, the mean (±SD) uric acid score was 4.24 (±0.61) mg/dL, and the mean (±SD) creatine kinase score was 98.29 (±63.01) U/L. In the OSMF group, the mean (±SD) urea score was 20.50 (±5.18) mg/dL, the mean (±SD) uric acid score was 4.03 (±0.65) mg/dL, and the mean (±SD) creatine kinase score was 95.25 (±63.40) U/L. In the group with palatal changes in reverse smokers, the mean (±SD) urea score was 21 (±8.91) mg/dL, the mean (±SD) uric acid score was 4.40 (±0.56) mg/dL, and the mean (±SD) creatine kinase score was 100.25 (±18.87) U/L. In the oral cancer group, the mean (±SD) urea score was 22.51 (±3.86) mg/dL, the mean (±SD) uric acid score was 4.20 (±0.85) mg/dL, and the mean (±SD) creatine kinase score was 113.76 (±106.38) U/L. In controls, the mean (±SD) urea score was 13.90 (±3.18) mg/dL, the mean (±SD) uric acid score was 4.71 (±0.85) mg/dL, and the mean (±SD) creatine kinase score was 75.70 (±13.07). The total mean (±SD) urea score was 20.87 (±5.67) mg/dL, the total mean (±SD) uric acid score was 4.30 (±0.77) mg/dL, and the total mean (±SD) serum creatine kinase score was 104.73 (±81.53). There were no statistically significant mean uric acid and creatine kinase levels.

## Discussion

Oral precancer, a condition of the oral mucosa linked to an elevated risk of malignancy, when detected and treated, has a better prognosis than late-stage malignancy [1]. The beginning and development of multistage carcinogenesis are significantly influenced by reactive species. The patient’s serum contains antioxidants that act as a line of defense against these reactive species. Cancer develops as a result of DNA damage and mutations when this equilibrium is disturbed [10]. People who use tobacco products have a much higher risk of developing renal disease than those who do not. The harmful ingredients in tobacco smoke are nicotine, tar, and carbon monoxide. Increased blood urea levels are associated with tobacco use [11,12], and tobacco use is a common etiological factor in these diseases. This study measured blood urea, uric acid, and creatine kinase levels to assess oral cancer and other potentially harmful conditions of the oral cavity. In contrast to the studies by Ara et al. [13] and Lawal et al. [14], the average age at which our patients were diagnosed with oral cancer was much younger in our cohort. This may be because of a rise in tobacco use among Indian youth. According to Ara et al. [13], however, most cases are seen in people over the age of 40 who are more predisposed to malignancy. This could be due to a number of risk factors, including the fact that as people age, their cells are exposed to carcinogens for longer periods of time.

Both Ara et al. [13] and Burgaz et al. [15] discovered that oral cancer disproportionately affected men. Oral cancer risk factors include tobacco use (smoking, chewing tobacco, and drinking), which may operate alone or together to increase that risk. In individuals who reverse smoke, however, the gender ratio is flipped, with more women than men engaging in this behavior. Ramesh et al. [16] conducted a study with female predominance in reverse smokers and have suggested various factors for an increase in female predilection, including the desire to keep their smoking habits hidden from their husbands, the increased likelihood of smoking chutta due to strong winds or splashing of water during household work, the desire to prevent hot ashes from falling on children, clothes, etc., and the desire to treat tooth pain in a culturally accepted way.

Between all three groups, urea levels rose significantly (P = 0.0001). This finding was consistent with that of Naik et al. [6] and Deepak et al. [2], who also found increased blood urea levels in OSMF patients and concluded that changes in biochemical values occur in OPMDs, but without a statistically significant P-value. Serum urea levels were significantly higher among smokers, which corroborated the findings of the research by Bn et al. [17]. They argue that the carcinogenic elements in cigarettes lead to cell death in the liver and kidneys, which may eventually lead to cancer. Consistent with earlier research, the current investigation linked OPMDs and oral cancer to increased blood urea levels. Patients in the research group had a history of using both smoked and smokeless tobacco; therefore, the same hypothesis might be applied to the present investigation. Serum urea levels were found to be considerably higher in OPMDs than in controls and much higher in oral cancer patients.

The results of the current investigation were in agreement with earlier studies by Ara et al. [13] and Lawal et al. [14], which showed that serum uric acid levels were significantly lower in cases of oral cancer. They proposed the following theories: free radicals cause toxicity, which is the main cause of cancer; uric acid protects against malignancy by inhibiting the formation of free radicals; and low serum uric acid in patients with oral cancer may be caused by interleukin-6 (IL-6) and tumor necrosis factor (TNF) secreted in patients with malignancy. Serum uric acid levels were significantly lower in lichen planus patients than in controls, which was consistent with research on potentially cancerous diseases by Shiva et al. [18] and Sree et al. [19]. They discovered that uric acid functions as a potent antioxidant and free radical scavenger in human bodily fluids. As a result of increased production of reactive oxygen and nitrogen species, oxidative stress, and an imbalance between reactive species, oxidative stress, and antioxidants, they came to the conclusion that low serum uric acid levels may contribute to the pathogenesis of OLP.

Hyperuricemia was shown to be a significant finding in patients with lichen planus, with controls having greater levels of antioxidants than cases, as was previously documented in earlier investigations by Gupta et al. [7]. According to research by Deepak et al. [2], released IL-6 and TNF may be responsible for lower levels of uric acid in the blood of patients with OPMDs and oral cancer, which may lead to an appetite deficit and malnutrition. Smokers had lower blood uric acid levels, according to research by Hanna et al. [20]. They argue that smoking causes more oxidative stress. One of the main etiological factors in OPMDs and oral cancer is the habit of smoking, and the decreased uric acid levels in the present study may be due to oxidative stress and the formation of oxygen radicals that cause the production of interleukin-1 (IL-1), IL-6, and TNF. However, Burgaz et al. [15] found elevated uric acid levels in individuals with oral cancer and hypothesized that this might be a consequence of the fast proliferation of malignant tissue and the enhanced nucleic acid turnover that this causes. These results suggest that the release of creatine phosphokinase (CPK) in response to muscle fiber injury, which occurs mostly as a result of proteolysis, indicating myofibrillar lysis, may be a factor in the development of mouth cancer and other potentially malignant conditions. These myofibrillar alterations may indicate cell damage, but not necrosis or permanent cell death. Proteolysis-inducing agents include IL-1, IL-6, and TNF. These cytokines are upregulated in premalignant and malignant states owing to localized synthesis by the lesional epithelium or by T-lymphocyte activation in connective tissue. Therefore, mouth lesions that are high in cytokines may exacerbate muscle injury in the subcutaneous connective tissue.

In addition, Joseph et al. [21] discovered elevated CPK levels in lichen planus patients and proposed that the difference in outcomes between cases and control subjects might be due to tissue damage that takes time to manifest. Additionally, it was stated that its existence is related to both the subject’s humoral state and the severity of the damage. In patients with oral submucous fibrosis, Joseph et al. [21] reported similar findings. They noted a slight difference in mean values between cases and controls but determined that this was not statistically significant. They discovered that after muscle damage, blood levels of creatine phosphokinase may change. Proteolysis is the primary cause of muscle injury. Submucous fibrosis patients have an increase in IL-I, IL-6, and TNF, all of which contribute to proteolysis. The presence of activated T-lymphocytes in premalignant lesions causes an increase in these cytokines. This finding agreed with that of Joseph et al. [21], who found that leukoplakia patients had higher CPK levels. They hypothesize that injury to the muscular layer of the implicated buccal mucosa might account for any observed fluctuation in the serum’s CPK value. Oral leukoplakia and CPK were not consistently linked, which may be because muscle damage is less severe in leukoplakia than in other lesions such as OSMF or because the pathophysiology of oral leukoplakia differs from that of OSMF, in which more muscle fibers are involved.

The research has certain limitations. Due to resource and time limitations, the sample size is modest, and urea, uric acid, and creatine kinase levels were not compared between variants and degrees of leukoplakia, lichen planus, OSMF, and palatal alterations in reverse smokers.

## Conclusions

Evaluating these measures before, during, and after treatment may be beneficial to further elucidate the role that urea, uric acid, and creatine kinase play in the treatment of oral cancer. Therefore, biochemical assessment is an essential component of the diagnostic process for patients who are suffering from OPMDs as well as oral cancer. The study’s findings showed that when patients with OPMD and oral cancer were compared to healthy controls, serum levels of urea and creatine kinase were found to be higher, whereas levels of uric acid were found to be lower. As a result, urea, uric acid, and creatine kinase may play a role as prognostic indicators of oral cancer and OPMDs.
